# Intrafamilial Disease Heterogeneity in Primary Hyperoxaluria Type 1

**DOI:** 10.1016/j.ekir.2024.07.026

**Published:** 2024-07-31

**Authors:** Lisa J. Deesker, Hazal A. Karacoban, Elisabeth L. Metry, Sander F. Garrelfs, Justine Bacchetta, Olivia Boyer, Laure Collard, Arnaud Devresse, Wesley Hayes, Sally-Anne Hulton, Cristina Martin-Higueras, Shabbir H. Moochhala, Thomas J. Neuhaus, Jun Oh, Larisa Prikhodina, Przemyslaw Sikora, Michiel J.S. Oosterveld, Jaap W. Groothoff, Giorgia Mandrile, Bodo B. Beck

**Affiliations:** 1 Department of Pediatric Nephrology, Emma Children’s Hospital, University of Amsterdam, Amsterdam, the Netherlands; 2 Centre de Référence des Maladies Rénales Rares, Hospices Civils de Lyon and Université Claude-Bernard Lyon 1, INSERM 1033 Unit, Lyon, France; 3 Néphrologie Pédiatrique, Centre de Référence MARHEA, Institut Imagine, Université Paris Cité, Hôpital Necker-Enfants Malades, Paris, France; 4 Department of Pediatrics, Centre Hospitalier Universitaire de Liège, Belgium; 5 Department of Nephrology, Cliniques universitaires Saint-Luc, Brussels, Belgium; 6 Department of Nephrology, Great Ormond Street Hospital for Children NHS Foundation Trust, London, UK; 7 Department of Nephrology, Birmingham Women’s and Children’s Hospital NHS Foundation Trust, Birmingham, UK; 8 Institute of Biomedical Technology, CIBERER, University of Laguna, San Cristóbal de La Laguna, Spain; 9 UCL Department of Renal Medicine, Royal Free Hospital, London, UK; 10 Department of Pediatrics, Children's Hospital Lucerne, Lucerne, Switzerland; 11 Department of Pediatric Nephrology, Medical University Medical Center Hamburg-Eppendorf, Hamburg, Germany; 12 Department of Inherited and Acquired Kidney Diseases, Veltishev Research and Clinical Institute for Pediatrics and Pediatric Surgery of the Pirogov Russian National Research Medical University, Moscow, Russia; 13 Department of Pediatric Nephrology, Medical University of Lublin, Lublin, Poland; 14 Genetic Unit and Thalassemia Center, San Luigi University Hospital, Orbassano, Italy; 15 Institute of Human Genetics, Center for Molecular Medicine Cologne, University Hospital of Cologne, Cologne, Germany; 16 Center for Rare and Hereditary Kidney Disease Cologne, University Hospital of Cologne, Cologne, Germany

**Keywords:** discordance, families, intrafamilial heterogeneity, kidney failure, PH1, primary hyperoxaluria

## Abstract

**Introduction:**

Primary hyperoxaluria type 1 (PH1) is known for its variable clinical course, even within families. However, the extent of this heterogeneity has not been well-studied. We aimed to analyze intrafamilial clinical heterogeneity and disease course among siblings in a large cohort of familial PH1 cases.

**Methods:**

A retrospective registry study was performed using data from OxalEurope. All PH1 families with 2 or more affected siblings were included. A 6-point PH1 clinical outcome scoring system was developed to grade heterogeneity within a family. Intrafamilial clinical heterogeneity was defined as a score ≥2. Kaplan-Meier analyses were used to analyze differences in kidney survival between index cases and siblings.

**Results:**

We included 88 families, encompassing 193 patients with PH1. The median interquartile range (IQR) follow-up time was 7.8 (1.9–17) years. Intrafamilial clinical heterogeneity, as defined by our score, was found in 38 (43%) PH1 families. In 54% of the families, affected siblings had a better outcome than the index case. Clinically asymptomatic siblings at the time of their diagnosis had a significantly more favorable clinical outcome based on the authors’ scoring system than siblings with clinical signs and index cases (*P* < 0.001). Kaplan-Meier analyses revealed that index cases reached kidney failure at an earlier age and earlier in follow-up compared to siblings (*P* < 0.001).

**Conclusions:**

Intrafamilial clinical heterogeneity was found in a substantial number of familial PH1 cases. Compared to index cases, siblings had significantly better clinical outcomes and kidney survival; thereby supporting the policy of family screening to diagnose affected siblings early to improve their prognosis.

## Graphical abstract

**Figure:**
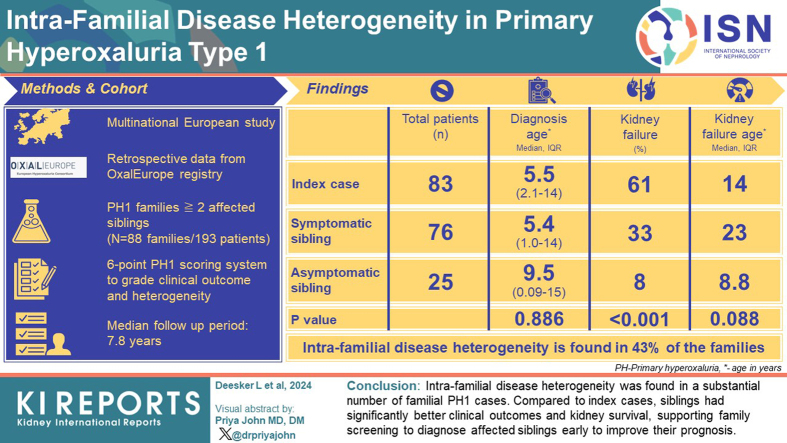

PH1 is a rare autosomal recessive metabolic disorder primarily affecting the kidneys.^1^ It is caused by a deficiency of the liver-specific enzyme alanine-glyoxylate aminotransferase, leading to endogenous oxalate overproduction. PH1 can manifest at any age with variable clinical expressivity, ranging from infants presenting with kidney failure (a condition referred to as infantile oxalosis) to minimally symptomatic adults with late kidney failure up to the seventh decade of life.^2^^,^^3^ Over 200 causative variants have been identified in the gene encoding for alanine-glyoxylate aminotransferase (*AGXT*), associated with various effects on the enzyme.^4^ Although some common missense variants resulting in alanine-glyoxylate aminotransferase mistargeting are associated with sensitivity to pyridoxine (a form of vitamin B6) supplementation and later onset of kidney failure, having such a variant is no guarantee for a good outcome.^5^^,^^6^ There are indications that even patients with identical *AGXT* variants within 1 family may have different clinical outcomes; however, hard data on this assumption are lacking so far.

In PH1, family screening is recommended. This is based on the high *a priori* risk of PH1 of 25% for siblings of an index case and the assumption that affected siblings of an index case will benefit from early diagnosis and rapid initiation of targeted treatment.^4^^,^^7^^,^^8^ However, conflicting data regarding the effectiveness of family screening have been reported in literature, and akin to having a favorable variant, early diagnosis is no guarantee for a good outcome.^9^

The recent introduction of RNA interference (RNAi) therapy has changed the therapeutic landscape for patients with PH1 because it lowers oxalate production in patients independent of *AGXT* genotype.^1^^,^^10^ Given the costs, unknown long-term outcomes, and side effects, RNAi treatment requires personalized application in patients with PH1. Insight into heterogeneity and the impact of family screening within PH1 could serve as important information for clinical decision making. Even more, the increasing use of RNAi treatment will make it difficult to assess intrafamilial clinical heterogeneity in the future, making it an important matter to be assessed at this time.

Unfortunately, the phenomenon of intrafamilial clinical heterogeneity has only been studied in reports on individual families, but has yet to be assessed systematically.^11^, ^12^, ^13^, ^14^, ^15^, ^16^, ^17^, ^18^, ^19^, ^20^, ^21^ In addition, no (common) definition exists of what the term heterogeneity in PH1 exactly means and how persistent the finding of diverse expressivity of disease is over time. This situation hampers clinical decision-making. Therefore, we set out to evaluate intrafamilial clinical heterogeneity among familial PH1 cases from the OxalEurope Registry by using a scoring system to grade the level of heterogeneity and evaluate the disease course of siblings based on simple criteria.

## Methods

A retrospective registry study was conducted analyzing data from the OxalEurope Registry, a European, institutional review board–approved database including data of 972 patients with PH1. Verification of PH diagnosis by genetic analysis, high clinical suspicion with abnormal metabolites, or liver biopsy is a prerequisite for inclusion into the OxalEurope database. All families with PH1 in the OxalEurope registry were identified. Index cases and their siblings (and thus not parents or cousins) were included in the study unless data regarding kidney failure were missing. Patients lost to follow-up were included in the study if data regarding kidney function at the time of diagnosis were available. If available, medical charts were reviewed for data validation and, in families with heterogeneity, possible determinants influencing clinical outcomes were reviewed.

Kidney failure was defined as either an estimated glomerular filtration rate of <15 ml/min per 1.73 m^2^, computed by the (modified) Schwartz formula^22^ for patients under 18 years, and by the Modification of Diet in Renal Disease formula^23^ for adult patients, or the need for dialysis. Patients with biallelic c.454T>A (p.Phe152Ile) or c.508G>A (p.Gly170Arg) variants of the *AGXT* gene were deemed vitamin B6–responsive by default based on the literature because data on individual biochemical response were not available.^4^^,^^5^^,^^24^ Total follow-up was calculated by subtracting the age at diagnosis from the age at last follow-up. Given that increased oxalate production is present from birth in PH1, age at last follow-up was also considered as an additional follow-up outcome in this study.

**Figure 1:**
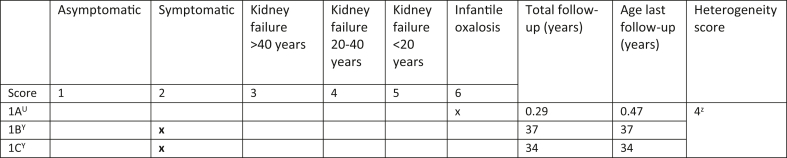
Six-point PH1 clinical outcome scoring system for calculating intrafamilial clinical heterogeneity. Here, an example is given of 1 family within the study. PH1, primary hyperoxaluria type 1; U, index case; Y, siblings of the index case; Z, heterogeneity score of family 1 (6 − 2 = 4).

A 6-point PH1 clinical outcome scoring system to express heterogeneity within a family was formulated and approved by a group of 4 experts in the field of hyperoxaluria (LJD, ELM, JWG, and BBB) after detailed examination of all Dutch PH1 families in the OxalEurope database (Figure 1). The clinical outcome of index cases and their affected sibling(s) at last known follow-up was graded using the following clinical outcome score: (i) asymptomatic; (ii) clinical signs without kidney failure, including nephrolithiasis and/or nephrocalcinosis; (iii) kidney failure above the age of 40 years; (iv) kidney failure between 20 and 40 years of age; (v) kidney failure before the age of 20 years; and (vi) infantile oxalosis (kidney failure before the age of 1 year). In the scoring system, a distinction was made for age at onset of kidney failure, given the differences in clinical and therapeutical consequences. The heterogeneity score within a family was calculated by subtracting the lowest score from the highest score among affected siblings. Significant intrafamilial clinical heterogeneity was defined as a score ≥2. As potential determinants of heterogeneity, we analyzed the following clinical characteristics: other comorbidities or inherited kidney abnormalities, vitamin B6 sensitivity, family screening with initiation of preventive treatment for siblings, and episodes of dehydration (including illness or neglect of conservative therapy).

For subgroup analysis, we divided the total cohort into the following 3 subgroups: (i) index cases, (ii) siblings with clinical signs at the time of diagnosis (e.g., nephrolithiasis, nephrocalcinosis, or kidney failure) referred to as symptomatic, and (iii) asymptomatic siblings without clinical signs at the time of diagnosis. Patients in the second group had been diagnosed by family screening or diagnostic evaluation following symptoms. Subsequently, analyses were performed to assess the impact of family screening on clinical outcomes between these groups. We excluded siblings from subgroup analysis if it was unclear whether they were symptomatic at the time of diagnosis. In addition, families were excluded from subgroup analysis if it was unknown who the index case was.

Numerical results are presented as medians with IQR, and categorical results as numbers and percentages. The Fisher-Freeman-Halton exact test, Mann-Whitney U test, and Kruskal Wallis test were used for statistical testing. Kaplan-Meier analysis was used to calculate kidney survival, counting kidney failure as an event either by age or follow-up time, as defined above. Log-rank test was used for comparison between groups. All tests were performed 2-sided, with a *P* < 0.05 considered statistically significant, using the latest version of SPSS (version 28, IBM Corp., Armonk, NY), R studio version 1.4.1106 (RStudio PBC, Boston, MA) and GraphPad Prism version 8 (GraphPad Software, San Diego, CA).

## Results

### Clinical Characteristics

#### Families

We identified 101 families with PH1 in the OxalEurope database. Thirteen families were excluded because of missing data, yielding a total of 193 patients with PH1 from 88 families (Supplementary Figure S1), all living in Europe (France, *n* = 23; Germany, *n* = 19; United Kingdom, *n* = 18; Netherlands, *n* = 15; Italy, *n* = 5; Spain, *n* = 2; Belgium, *n* = 2; Poland, *n* = 2; Russia, *n* = 1; and Sweden, *n* = 1). For 26 families, their country of origin was outside Europe (Algeria, *n* = 1; Albania, *n* = 1; Bangladesh, *n* = 1; Iraq, *n* = 1; Jordan, *n* = 1; Kuwait, *n* = 1; Lebanon, *n* = 2; Morocco, *n* = 4; Pakistan, *n* = 6; Syria, *n* = 1; Tunisia, *n* = 2; and Turkey, *n* = 5). The median number of siblings per family was 2; 16 families had more than 2 affected siblings.

Family screening had been conducted in 77% of families (*n* = 64). Family screening was not performed in 7 cases, either due to short time since diagnosis of the index case or because a sibling had already developed symptoms at the time of diagnosis of the index case. In other families, screening had not been performed against medical advice (*n* = 2) or for unknown reasons (*n* = 10). Regarding the latter, it was notable that in 6 out of 10 families, the index case had been diagnosed before 1990. At last follow-up, at least 1 case of kidney failure had occurred in 59 (67%) families and in 22 (29%) families 1 or more patients had died. Detailed family characteristics are presented in Table 1.

**Table 1:** Clinical characteristics of families with PH1

Characteristics of families	Value	
	*n*/total	%
Total number of families	88	
Follow-up ≥15 yr since birth	56/88	64
Follow-up ≥15 yr since diagnosis	23/88	26
VB6–responsive *AGXT* variants (c.454T>A and c.508G>A) Homozygous variants/heterozygous variants	37/85 21/85 16/85	44 25 19
Family screening	64/83	77
Index cases with older sibling(s) at diagnosis	30/88	34
Siblings treated with conservative treatment <6 mo after birth	19/88	22
Family members with kidney failure	59/88	67
Deceased family members	22/76	29
Clinical outcome of sibling milder than in index case^a^	43/79	54

IQR, interquartile range; KF, kidney failure; PH1, primary hyperoxaluria type 1; VB6, vitamin B6.

Values are expressed as *n* with percentage of (sub)group.

a Based on a lower score on the clinical outcome scoring system compared to the index case.

Information regarding the prevalent *AGXT* variant was missing for 3 families. The other 85 families carried 41 different disease-causing variants in the *AGXT* gene. The 4 most prevalent biallelic pathogenic variants were c.508G>A (*n* = 16; 19%), c.33dupC (*n* = 7; 8.2%), c.731T>C (*n* = 6; 7.1%) and c.454T>A (*n* = 5; 5.9%). In addition, 37 of the 85 families (44%) were homozygous (*n* = 21; 25%) or heterozygous (*n* = 16; 19%) for a B6-responsive variant.

In approximately half of the families (*n* = 43; 54%), affected siblings had a better clinical outcome than the index case according to the scoring system (i.e., a lower score). In only 4 of these families (9%), a second sibling had an equal or higher clinical outcome score than the index case. In addition, in 35% (*n* = 28) of all families, affected siblings and index cases had equal scores, with a median of 2.86 (range 1.33–6) per family.

#### Individuals

**Figure 2:**
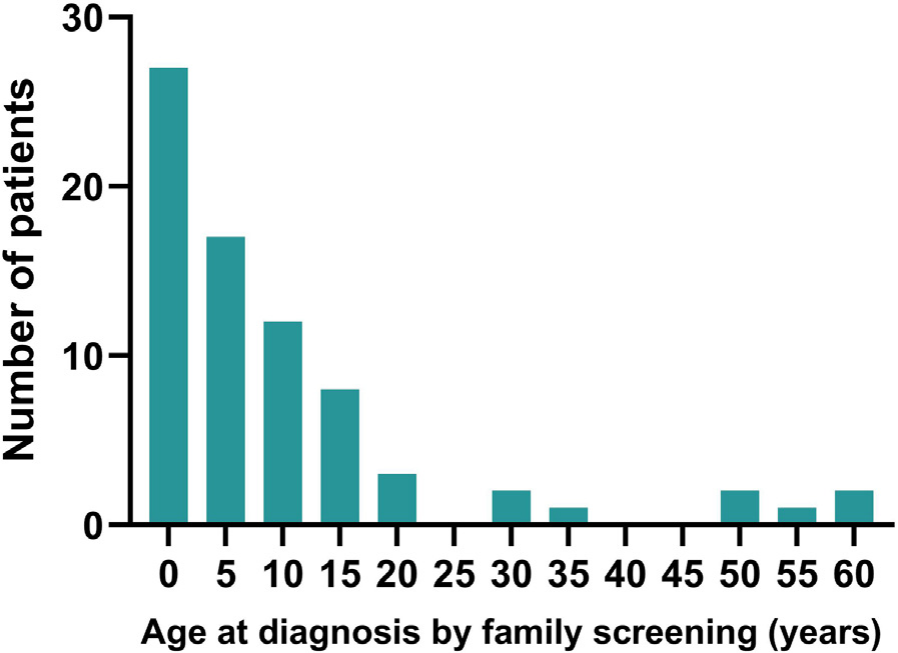
The number of patients diagnosed by family screening by age at diagnosis. Each bar represents a 5-year range.

We included 193 patients with PH1. Information regarding follow-up after diagnosis was unavailable for 7 patients (3.6%). The median (IQR) follow-up time of all patients was 7.8 (1.9–17) years and median age at last follow-up was 18 (8.7–35) years. Patients were diagnosed at a median age of 5.7 (1.0–14) years. We identified 83 patients as index cases (group 1 in Table 2) who were diagnosed at a median age of 5.5 (2.1–14) years. At the time of diagnosis, 35 (42%) had already developed kidney failure. Seventy-seven patients (siblings of index cases) had been diagnosed by family screening; 25 out of 74 (34%) patients with information available were asymptomatic at the time of screening (group 3 in Table 2). Siblings diagnosed by family screening had a median age of 4.9 (0.1–13) years, with a range of 0.0 to 59 years (Figure 2). The noticeable outliers of siblings who were diagnosed by family screening at about the age of 50 years resulted from an index case being diagnosed late in adulthood due to the sudden onset of kidney failure as presenting symptom. None of the patients in the cohort were treated with RNAi therapy. In addition, no patients were diagnosed via prenatal screening.

**Table 2:** Characteristics of index cases and siblings

Characteristics and outcome	Index cases	Siblings symptomatic at diagnosis	Siblings asymptomatic at diagnosis	*P*-value
Number of patients, *n*	83	76	25	N/A
Follow-up time, median (IQR)	7.2 (2.0–17)	9.3 (3.2–17)	7.4 (1.8–16)	0.539^a^
Age at diagnosis, median (IQR)	5.5 (2.1–14)	5.4 (1.0–14)	9.5 (0.09–15)	0.886^a^
Clinical outcome score^b^, median (IQR)	4.0 (2.0–5.0)	2.0 (2.0–3.8)	1.0 (1.0–1.5)	<0.001^a^^,^^c^
Kidney failure, *n* (%)	51/83 (61)	25/76 (33)	2/25 (8)	<0.001^d^
Age kidney failure, median (IQR)	14 (0.9–25)	23 (14–44)	8.8	0.088^a^
Deceased patients *n* (%)	17/79 (20)	6/70 (8.6)	2/21 (9.5)^e^	0.077^d^
Age at death, median (IQR)	14 (4.2–24)	22 (1.1–34)	43	0.553^a^

IQR, interquartile range; N/A, not applicable; PH1, primary hyperoxaluria type 1.

Age or time is expressed in years. Values are expressed as *n* with percentage of subgroup and as median with IQR.

a Kruskal Walli’s test.

b Score based on the 6-point PH1 clinical outcome scoring system.

c *Post hoc* analysis between the symptomatic and asymptomatic sibling groups performed with Mann-Whitney U test (*P* < 0.001).

d Fisher-Freeman-Halton exact test.

e One of the 2 deceased patients among the siblings asymptomatic at time of diagnosis died at the age of 74 years for reasons unknown.

### Heterogeneity Score of PH1 Families

**Figure 3:**
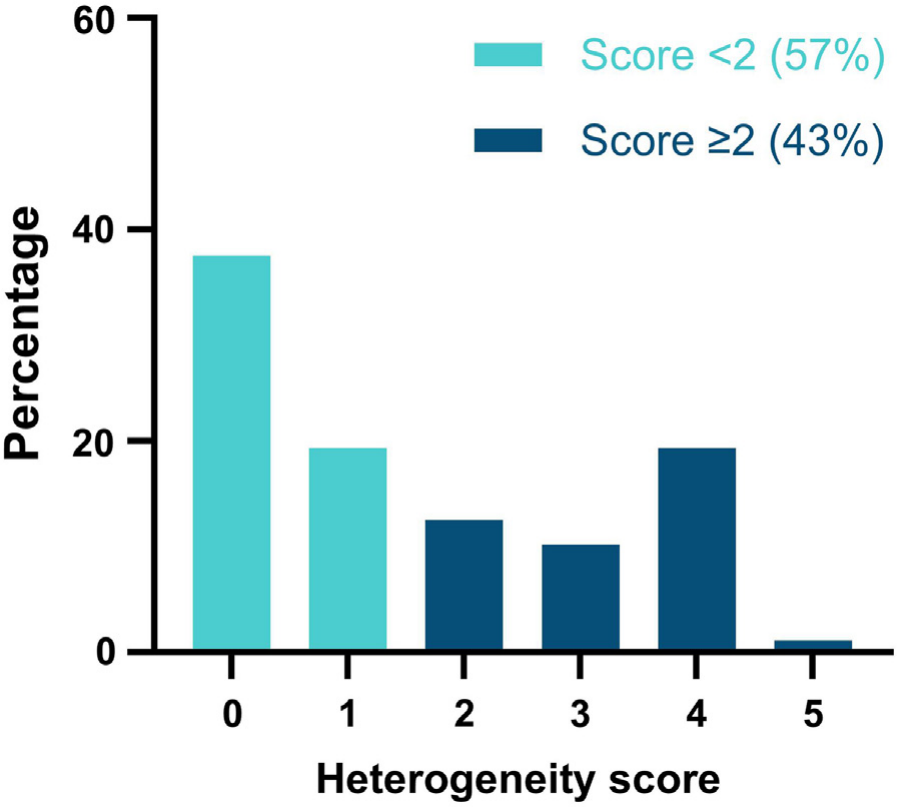
The distribution of heterogeneity score of all 88 families. For each family member with PH1, the clinical outcome was graded using a 6-point PH1 score. The heterogeneity score of each family was then calculated by subtracting the lowest score from the highest score among all siblings. PH1, primary hyperoxaluria type 1.

Heterogeneity, defined as a score of ≥2 points, was present in 43% (*n* = 38) of all families (Figure 3). In addition, 27 families (31%) had a score of ≥3 points and 18 (20%) scored ≥4 points. Only 1 family (1.1%) had a heterogeneity score of 5 points (e.g., a case of infantile oxalosis and an asymptomatic sibling aged 18 years). Of the 23 families with a follow-up of more than 15 years since diagnosis, 11 (48%) had a heterogeneity score of ≥2 points. In addition, of the 56 families with a follow-up time of more than 15 years since birth, 26 (46%) had a heterogeneity score of ≥2 points. At the other end of the spectrum, we found 12 families with similar clinical outcomes among all siblings regarding age at onset of kidney failure.

#### Heterogeneity in Families With B6-Responsive AGXT Variants

**Figure 4:**
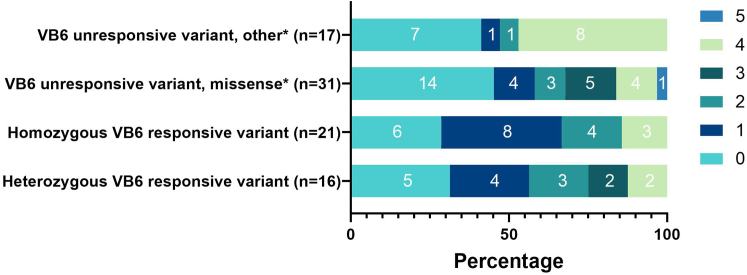
Distribution of heterogeneity scores in families with and without (partly) vitamin B6 (VB6) –responsive variants. Shown are the heterogeneity scores of families stratified by *AGXT* variant. Heterogeneity score is calculated by subtracting the lowest score from the highest score among the siblings, using the 6-point PH1 clinical outcome scoring system. The number of families with a specific heterogeneity score is noted in the boxes. PH1, primary hyperoxaluria type 1. ∗The group “missense” encompasses all families with a homozygous missense *AGXT* variant. The group “other” includes mostly families with a homozygous null variant (*n* = 16) and 1 heterozygous missense/null variant.

The distribution of heterogeneity scores among families with vitamin B6–responsive (homozygous and heterozygous) and unresponsive *AGXT* variants is illustrated in Figure 4. The median (IQR) score was 1 (0–2) in the homozygous and heterozygous vitamin B6–responsive groups, and 1 (0–4) in the group completely unresponsive to vitamin B6 (*P* = 0.82, Mann-Whitney U test). However, 8 out of 17 families (47%) with a homozygous or heterozygous B6-unresponsive null variant had a heterogeneity score of ≥4 points compared to 3 out of 21 families (14%) with a homozygous vitamin B6–responsive variant (*P* = 0.035).

#### Determinants Influencing Clinical Outcome

We searched for possible clinical determinants and confounding factors influencing outcomes in all 38 families with clinical heterogeneity (score ≥2). Insufficient information was available regarding possible determinants of heterogeneity for 6 of 38 families (16%). In 20 of the remaining 32 families (63%), a potential explanation for the intrafamilial heterogeneity was found; in 15 families’ medical interventions (e.g., prompt initiation of vitamin B6 supplementation following PH1 diagnosis by family screening) could explain clinical heterogeneity; and in 5 families, the index case had been exposed to a possible trigger before presenting with kidney failure. These potential triggers included an episode of gastrointestinal symptoms (*n* = 3), alcohol abuse (*n* = 1), and a congenital kidney defect specified as unilateral kidney dysplasia (*n* = 1).

In 10 of 32 families (31%), heterogeneity could not (plausibly) be explained by external factors or triggers (as defined in the methods section). In 8 families, siblings had started treatment (either with vitamin B6 or conservative measures) promptly after diagnosis. At the time of diagnosis of the index case, older siblings were either asymptomatic (*n* = 5) or were found to have nephrocalcinosis only (*n* = 3) while not receiving any treatment. Thus, clinical heterogeneity could not be adequately explained by timelier medical intervention. In the other 2 families, we could not find any potential influencing factors or explanations that might explain differences in clinical outcomes.

In 2 families with a high heterogeneity score and *AGXT* variants associated with vitamin B6 unresponsiveness, the younger sibling had been diagnosed by family screening directly after birth but still had a worse outcome (i.e., infantile oxalosis) than the index case. Accordingly, early diagnosis and medical intervention were unable to prevent infantile oxalosis.

### Clinical Outcome of Individuals (Index Cases Versus Siblings)

A total of 184 patients were included in the subgroup analysis (Supplementary Figure S1). No significant difference was found in total follow-up time between the groups. At last follow-up, the median (IQR) clinical outcome score of index cases was 4 (2–5) compared to a score of 2 (2–3.8) among siblings with clinical signs at the time of diagnosis and a score of 1 (1–1.5) among asymptomatic siblings at the time of diagnosis (*P* < 0.001, Table 2). Kidney failure, at the time of diagnosis or during follow-up, was more frequent among index cases (61%) than among siblings with clinical signs at time of diagnosis (33%) and siblings who were asymptomatic at time of diagnosis (8%) (*P* < 0.001).

**Figure 5:**
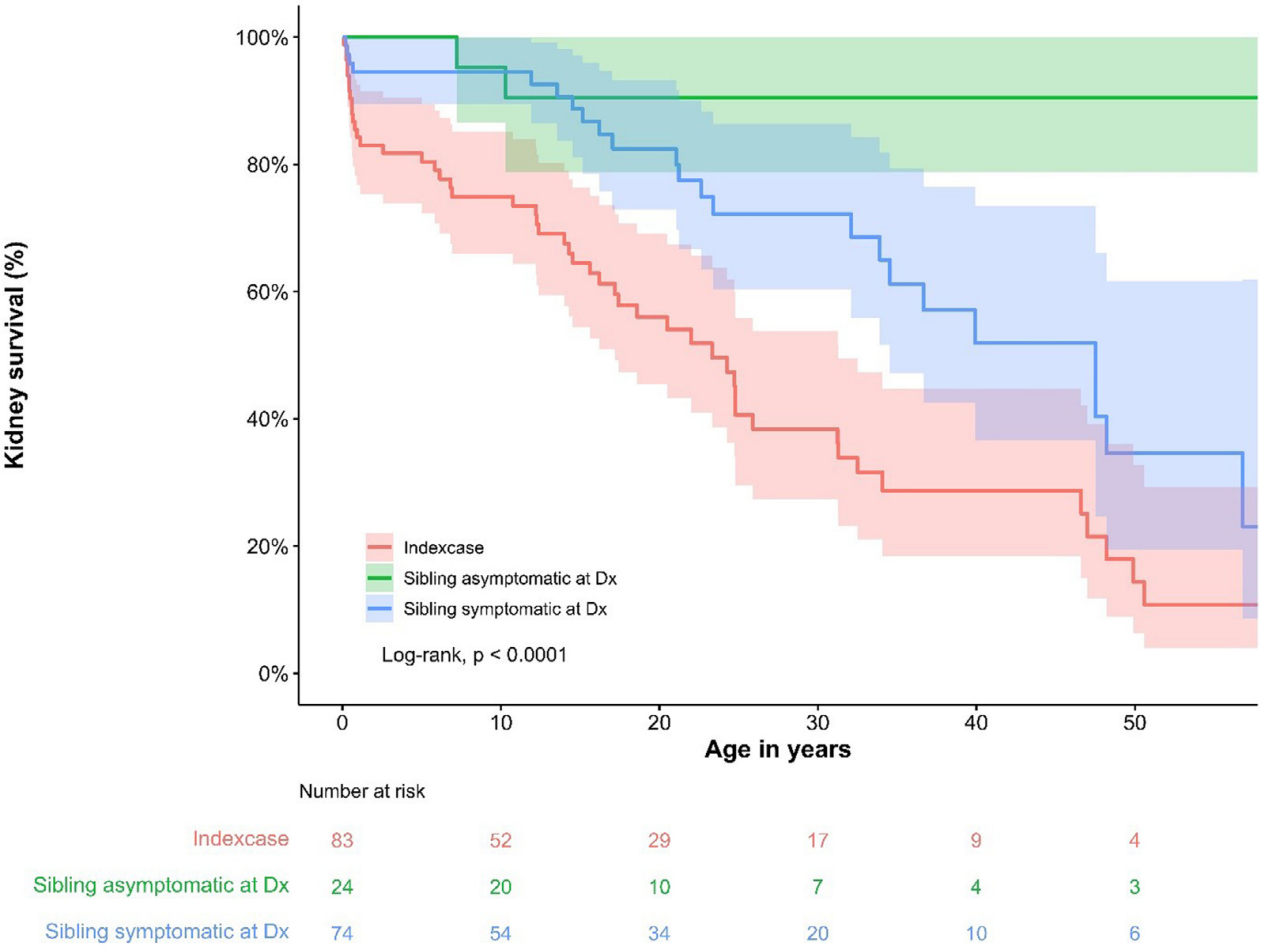
Kaplan-Meier analysis including confidence bands of death-censored kidney survival by age, stratified by index case, siblings asymptomatic at time of diagnosis and siblings symptomatic at time of diagnosis. Log-rank test analysis showed a significant difference (*P* < 0.001) between the groups. Dx, diagnosis.

**Figure 6:**
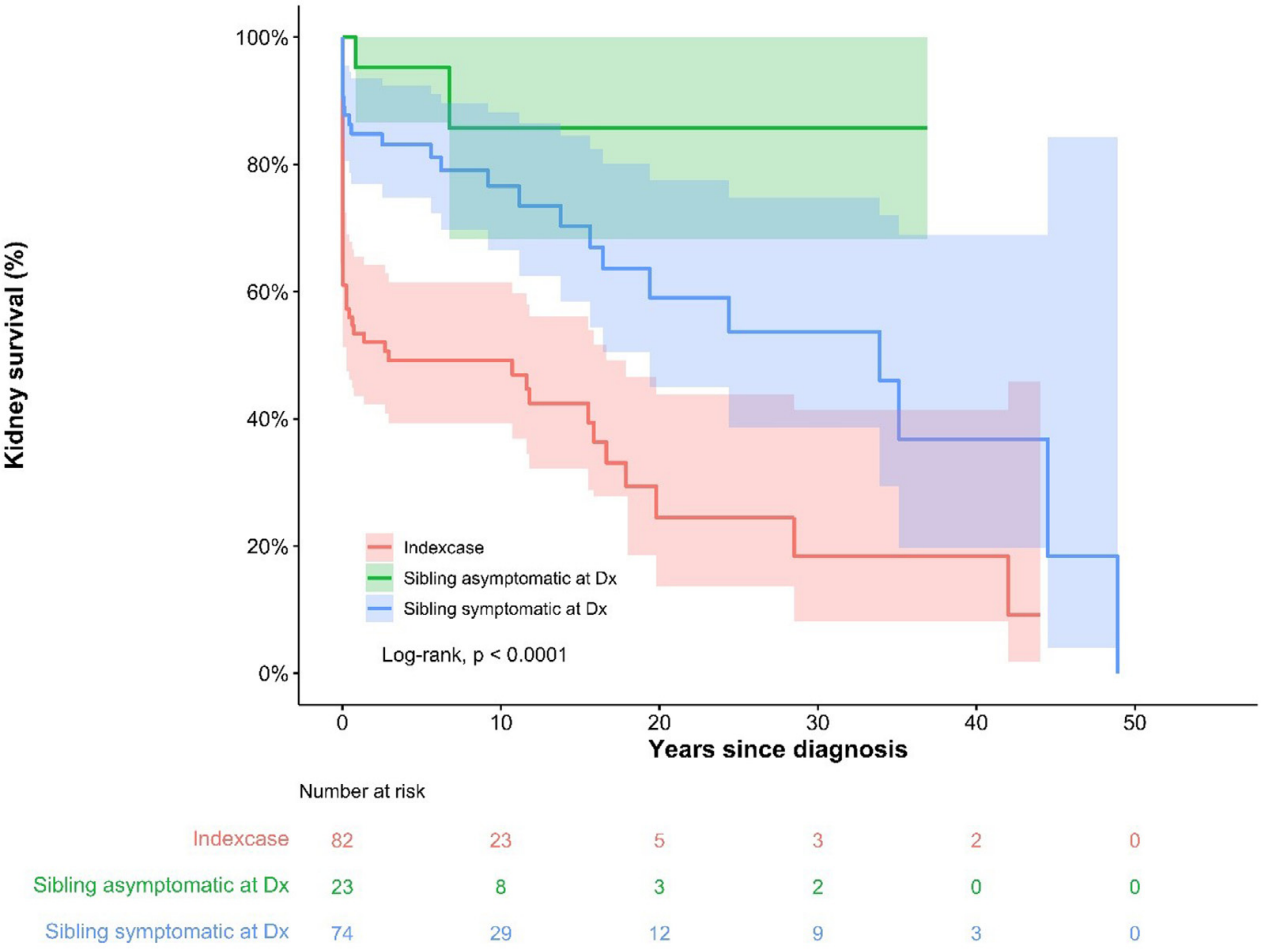
Kaplan-Meier analysis of death-censored kidney survival by years since diagnosis, stratified by index case, siblings symptomatic at the time of diagnosis, and siblings asymptomatic at the time of diagnosis. Log-rank test analysis showed a significant difference (*P* < 0.001) between the groups. Dx, diagnosis.

Kaplan-Meier analyses (Figures 5 and 6) revealed a significant difference in the death-censored kidney survival between index cases and sibling(s). Index cases reached kidney failure at an earlier age and earlier in follow-up compared to siblings (Log-rank, *P* < 0.001). Notably, death occurred more frequently among index cases (20%) than among siblings (8.6% and 9.5% among siblings with and without clinical signs at time of diagnosis, respectively); albeit this difference did not reach statistical significance (*P* = 0.077).

## Discussion

In this study, we describe the differences in clinical outcomes among siblings affected by PH1 from 88 families registered in the OxalEurope registry. We found significant heterogeneity in clinical outcome between affected family members with the same causative variant in 43% of families. This observed heterogeneity of the disease course could not always be explained by time of medical intervention or environmental factors. Families with B6-responsive variants had similar heterogeneity scores to those with B6-unresponsive variants. Still, the latter group showed a larger proportion of severe heterogeneity scores (i.e., ≥4 on a scale of 5). Siblings had a significantly better clinical outcome than index cases, especially those asymptomatic at diagnosis.

Our findings confirm that the significant intrafamilial clinical heterogeneity observed in previous reports of single families is real.^11^, ^12^, ^13^, ^14^, ^15^, ^16^, ^17^, ^18^, ^19^, ^20^, ^21^ Ours is the first study to describe this in a large cohort and to identify the extent of heterogeneity. Intrafamilial clinical heterogeneity seems to be a permanent rather than a temporary phenomenon, persisting throughout long follow-up periods. Previous reports have hypothesized different reasons for the clinical heterogeneity within the PH1 population. This may partly be explained by genotype-phenotype correlations, a hypothesis that holds true especially for vitamin B6–responsive variants.^5^

However, given the observed intrafamilial clinical heterogeneity, clinical heterogeneity within PH1 cannot entirely be ascribed to the type of *AGXT* genotype. The overall outcome was better in B6-responsive variants, and severe clinical heterogeneity was less significantly apparent in siblings with B6-responsive variants. Nevertheless, even in these families, extreme differences in outcome did occur. We had expected vitamin B6 responsiveness to associate with higher heterogeneity scores among families with vitamin B6–responsive *AGXT* variants due to effective and timely initiation of vitamin B6 therapy in siblings.^25^ However, we found no significant difference in intrafamilial clinical heterogeneity scores between these family groups (families with severe heterogeneity scores were more prevalent among carriers of vitamin B6–unresponsive variants). This may possibly be explained by the overall better clinical outcome found among vitamin B6–responsive patients (thus minimizing intrafamilial clinical heterogeneity) or possibly, noncompliance to vitamin B6, which was not evaluated in this study.

When looking into potential causes of clinical heterogeneity, it has been hypothesized that external environmental factors (including dietary habits and hydration) could at least partly explain heterogeneity. In our study we found some cases of intrafamilial clinical heterogeneity after an episode with gastrointestinal symptoms and dehydration, supporting that sporadic events may potentially have influence. However, for other factors the evidence is limited, and it may be assumed that most environmental factors are similar for siblings of 1 family, thus not explaining all clinical differences. Although some factors potentially related to clinical outcome, such as genotype, diet, socioeconomic status, and access to medical treatment, are better matchable in families, it is doubtful that this is the case for the unknown variables associated with discrepant phenotypic expressivity of PH1 disease.

Timely initiation of medical management is expected to influence clinical outcome positively. However, in 5 out of 38 heterogeneous families, elder asymptomatic siblings had a better outcome than the index case despite not receiving any treatment, suggesting that heterogeneity cannot be entirely explained by differences in medical management. Moreover, in 2 families, a younger sibling had been diagnosed by family screening shortly after birth and nevertheless developed infantile oxalosis (and thus had a more severe clinical outcome than the index case) despite early diagnosis and treatment. Therefore, the outcomes of our cohort with the combination of identical *AGXT* variants within families, similar external environments, and the above-mentioned findings regarding medical intervention suggest that other factors (i.e., genetic modifiers or other unidentified differences in glyoxylate metabolism) could play a role in the clinical heterogeneity within families.

Another important finding in this study is that siblings generally had significantly better clinical outcomes and kidney survival, supporting the idea that family screening may be beneficial in improving the prognosis of siblings, which is in line with current European practice recommendations.^1^^,^^4^ Conversely, previous research has reported that the clinical course of siblings diagnosed with PH1 by family screening is similar to that of index cases regarding metabolites and decline of kidney survival over time, indicating a similar disease severity.^9^ However, in this study, index cases with kidney failure (at the time of or within 60 days after diagnosis) were excluded, likely distorting the impact of family screening because kidney failure is the main adverse outcome in PH1 that one aims to prevent using family screening. When comparing our outcomes, we found that after the first drop in kidney survival (Figure 5), our results are like those mentioned above (indicated by a parallel decline of the index cases and symptomatic sibling curves).

Apart from the overall difference in outcome between index cases and siblings, our study also found notable differences in outcome between siblings diagnosed by family screening, with siblings asymptomatic at time of diagnosis having a better clinical outcome at last follow-up than siblings with clinical signs. Although the numbers are small and the design of our study does not allow to draw any conclusions regarding the reason for this difference in outcome and thus the impact of family screening, one might assume that early family screening, preferably before the onset of symptoms, may result in a better prognosis. Symptomatic siblings possibly had already developed some kidney damage (due to nephrocalcinosis or urolithiasis),^26^ resulting in less favorable outcomes than asymptomatic siblings. It should be noted that bias may be present, because older asymptomatic siblings may have had a more favorable natural course of disease than symptomatic siblings. However, our findings align with previous studies suggesting that early diagnosis and treatment initiation is beneficial in preventing kidney failure in siblings.^7^^,^^8^ We therefore advise early family screening, preferably by genetic testing, and prompt initiation of treatment. In an era with rapidly evolving treatment options, with availability of novel RNAi therapies in addition to conservative measures, this possible beneficial effect of family screening might be of special importance for the clinical outcome in the future.

Our study is the first to structurally examine intrafamilial clinical variability in PH1. To study intrafamilial heterogeneity, a scoring system based on reliable outcomes was necessary to define and reliably compare and analyze familial heterogeneity within PH1. Therefore, in line with previous studies on metabolic diseases, we created a scoring system based upon the (Markov) state-transition model, designed to depict prevalence and progression of disease. For the robustness of the scoring system, we included only well-reported and easy to score, clear outcome measures, leading to the current 6-point PH1 clinical outcome score. However, classifying such a heterogenic disease is complex, and nuances in clinical outcomes may have been lost. For instance, we did not include death in the scoring system because premature death may often be attributed to multiple factors, including transplantation complications. Neither was the stone rate included because this is often not reliably reported to medical professionals or captured in the patient record. Despite these limitations, we argue that the 6-point PH clinical outcome scoring system is representative of intrafamilial heterogeneity within families with PH1 and suitable to analyze this outcome in our cohort of families with PH1.

The retrospective design of our study has some limitations; follow-up duration differed in every family, and some data were missing. Therefore, additional information about the detailed disease course, including comparison of urinary oxalate levels, was not available for analysis. In addition, due to the limited number of families, type II errors (lack of statistical power to detect effects) may have occurred, reducing the reliability of certain analyses.

Based on our findings, we may conclude that intrafamilial clinical heterogeneity is common in PH1. The cause of this heterogeneity remains to be determined in most cases. Therefore, intrafamilial clinical heterogeneity should be more actively investigated in studies assessing clinical and environmental factors, genetic modifiers, and other pathways involved in glyoxylate metabolism. Our outcomes may support clinical care and provide insights for better individualized therapies.

## Disclosure

JWG and SFG have received an unconditional grant from both Alnylam Pharmaceuticals and Dicerna (Novo Nordisk) Pharmaceuticals to fund the OxalEurope Registry. The OxalEurope registry received financial support from Novo Nordisk for 3 years for epidemiological studies in patients with primary hyperoxaluria. SFG, JO, and AD declared consultancy fees from Alnylam. WH declares consultancy fees from Arbor Biotech and Alnylam. JB received consultancy fees from Alnylam, Novo Nordisk, and Biocodex; and speaker fees and research grants from Alnylam. OB received consultancy fees from Alnylam and Biocodex. SHM received consultancy fees from Alnylam, Dicerna, and Arbor Biotech. CM-H received consultancy fees from Novo Nordisk and Arbor Therapeutics. BBB and GM received consultancy fees from Alnylam and Novo Nordisk. BBB was supported by the intramural Köln-Fortune Programm der Medizinischen Fakultät. University Hopital of Cologne (grants KF472/2018 and KF183/2022). All the authors declared no competing interests.

## Supplementary Material

Supplementary File (PDF) **Figure S1.** Flowchart of family selection. **STROBE Statement**.

## Footnotes

Supplementary File (PDF)
**Figure S1.** Flowchart of family selection. **STROBE Statement**.
